# Graphene for gold extraction

**DOI:** 10.1093/nsr/nwac160

**Published:** 2022-08-17

**Authors:** Kostya S Novoselov

**Affiliations:** Institute for Functional Intelligent Materials, National University of Singapore, Singapore

Gold is a unique element, characterized not only by limited, though still reasonable, availability (useful for monetary applications) but also for its rather unique properties useful for many industries [1]: chemical inertness, high electrical conductivity, plasticity, etc. As gold reserves in Earth's crust diminish with traditional mining, ‘urban mining’ that recycles gold from gold-containing wastes (such as electronic waste, e-waste) has emerged as an important supply method for gold sustainability [2]. One critical step in such a recycling process is the use of an adsorbent to extract gold from e-waste leachate that contains complex metal elements. Therefore, the gold adsorbent should have a high extraction capacity, selectivity and, importantly, economic viability. Although many porous materials have been developed for gold extraction from e-waste [3–6], materials that show high gold extraction capacity with precise gold selectivity to ppm or even lower concentrations are highly required.

2D materials, especially graphene, have one of the highest specific surface area, essential for a high adsorption capacity. Early research on gold-doped graphene showed that graphene could chemically reduce aqueous gold ions to metallic gold driven by a redox reaction between graphene and gold ions [7,8]. Together, these two properties suggest that graphene could be a good candidate for gold extraction. However, possibly because of an intrinsic hydrophobicity, which offers graphene a poor dispersibility in aqueous gold solution and sacrifices its large surface area, the demonstration of graphene for gold extraction was lacking.

Recently, the research group led by Yang Su from Tsinghua Shenzhen International Graduate School, Hui-Ming Cheng from the Shenzhen Institute of Advanced Technology of the Chinese Academy of Sciences and Andre K. Geim from the University of Manchester discovered that reduced graphene oxide (rGO), a chemical derivative of graphene, extracts gold from e-waste with ultra-high capacity and very good selectivity [9]. The technology can be scaled up for industrial applications. After rGO is directly mixed with gold solution for a few minutes, the metallic gold is extracted and deposited on rGO. Notably, >95% of gold ions are reduced to metallic gold by rGO, which avoids the elution and precipitation necessary in post-adsorption processing. The rGO shows a gold extraction capacity of ∼1000 mg/g to 1 ppm gold solution, which is significantly higher than state-of-the-art gold adsorbents [3–6]. Even if the gold concentration is as low as 20 ppt, the rGO can achieve effective gold extraction. More interestingly, the rGO exclusively extracts gold without contamination of other 14 coexisting metallic elements generally seen in e-waste, manifesting excellent extraction selectivity.

**Figure 1.**
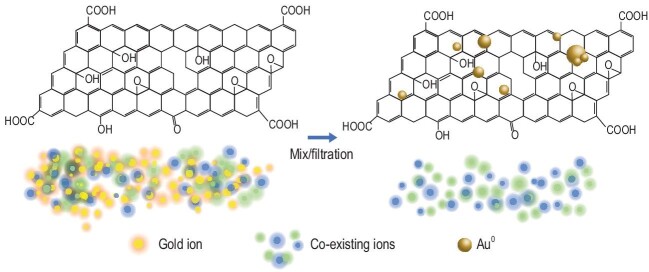
Reductive extraction of gold by rGO with ultra-high selectivity. By mixing rGO with a solution with gold ions present or filtrating such a solution through the rGO membrane, gold ions are reduced by rGO and deposited as metallic gold on rGO, without contamination of coexisting ions.

The heterogeneous structure of rGO plays a critical role in the reported ultra-high extraction capacity and precise selectivity (Fig. 1). The pristine graphene areas of rGO adsorb and reduce gold ions, similarly to gold ion doped graphene. The residual oxidized regions of rGO provide a good dispersibility so that graphene areas are readily available for gold extraction. This research further shows that the gold ions are adsorbed and reduced at the pristine graphene areas, while other coexisting metal elements are adsorbed at the oxidized regions via coordination, ion exchange, etc. Exploiting such site-specific adsorption by pristine graphene areas and oxidized regions, the coexisting metal ions can be stripped off by protonation of the oxidized regions without affecting the gold extraction happening on the graphene regions, hence efficient gold extraction can be achieved.

Interestingly, the wrinkled and wrapped areas of graphene tend to extract more gold than the flat areas, which is explained by a decreased adsorption energy barrier in these areas, suggesting the uniqueness of the monolayer graphene compared with its bulk counterparts, worth exploring in depth.

The reported gold extraction by rGO is highly promising for its practical e-waste recycling. The 1-gram rGO can extract 1.8 grams of gold (at 10-ppm concentration) and the price of commercially available GO is <1% of the price of gold. Considering the demonstrated scalability and developed continuous gold recycling process, the rGO is figuratively turning the waste into precious gold, like the philosopher's stone.

Using rGO for gold extraction from e-waste is unprecedented. The superior performance will open a novel, green and efficient route to address the challenges of gold sustainability and global e-waste accumulation. Fundamentally, the research shows that the fine-tuning of the atomic structure of 2D materials can modulate their interaction with target adsorbate(s). As adsorption is essential for many energy- and environmental-related processes, it opens an elegant and effective way for the development of 2D material-based adsorbents, which is a gold mine worthy of being intensively explored.

***Conflict of interest statement.*** None declared.
